# Quantifying Changes in the Language Used Around Mental Health on Twitter Over 10 Years: Observational Study

**DOI:** 10.2196/33685

**Published:** 2022-03-30

**Authors:** Anne Marie Stupinski, Thayer Alshaabi, Michael V Arnold, Jane Lydia Adams, Joshua R Minot, Matthew Price, Peter Sheridan Dodds, Christopher M Danforth

**Affiliations:** 1 Computational Story Lab Vermont Complex Systems Center University of Vermont Burlington, VT United States; 2 Advanced Bioimaging Center University of California Berkeley, CA United States; 3 Data Visualization Lab Khoury College of Computer Sciences Northeastern University Boston, MA United States; 4 Department of Psychological Science University of Vermont Burlington, VT United States; 5 Department of Computer Science University of Vermont Burlington, VT United States; 6 Department of Mathematics and Statistics University of Vermont Burlington, VT United States

**Keywords:** mental health, stigma, natural language processing

## Abstract

**Background:**

Mental health challenges are thought to affect approximately 10% of the global population each year, with many of those affected going untreated because of the stigma and limited access to services. As social media lowers the barrier for joining difficult conversations and finding supportive groups, Twitter is an open source of language data describing the changing experience of a stigmatized group.

**Objective:**

By measuring changes in the conversation around mental health on Twitter, we aim to quantify the hypothesized increase in discussions and awareness of the topic as well as the corresponding reduction in stigma around mental health.

**Methods:**

We explored trends in words and phrases related to mental health through a collection of 1-, 2-, and 3-grams parsed from a data stream of approximately 10% of all English tweets from 2010 to 2021. We examined temporal dynamics of mental health language and measured levels of positivity of the messages. Finally, we used the ratio of original tweets to retweets to quantify the fraction of appearances of mental health language that was due to social amplification.

**Results:**

We found that the popularity of the phrase *mental health* increased by nearly two orders of magnitude between 2012 and 2018. We observed that mentions of *mental health* spiked annually and reliably because of mental health awareness campaigns as well as unpredictably in response to mass shootings, celebrities dying by suicide, and popular fictional television stories portraying suicide. We found that the level of positivity of messages containing *mental health*, while stable through the growth period, has declined recently. Finally, we observed that since 2015, mentions of mental health have become increasingly due to retweets, suggesting that the stigma associated with the discussion of mental health on Twitter has diminished with time.

**Conclusions:**

These results provide useful texture regarding the growing conversation around mental health on Twitter and suggest that more awareness and acceptance has been brought to the topic compared with past years.

## Introduction

### Background

Recent estimates place 1 in 10 people globally as experiencing some form of mental illness [1], with 1 in 30 living with depression [2]. These rates put mental illness among the leading causes of ill health and disability worldwide. Moreover, rates of mental health disorders and deaths by suicide have increased in recent years, especially among young people [3].

Since the beginning of the COVID-19 pandemic and the subsequent social isolation brought on by lockdowns, stay-at-home orders, and the transition to remote work, there have been drastic declines in both physical and social activity, as well as increases in screen time and symptoms of depression [4]. Google searches for mental health–related topics increased in the first weeks of the pandemic, leveling out after more information regarding stay-at-home orders was released [5]. Since March 2020, there has also been a measured increase in suicidal ideation that is associated with increased feelings of social isolation [6]. The Crisis Text Line service reported receiving a higher-than-average volume of messages every day since March 16, 2020, with the main topics being anxiety, depression, grief, and eating disorders [7]. Price et al [8] also found that daily *doomscrolling*—repeatedly consuming negative news and media content on the web—was associated with same-day increases in depression and posttraumatic stress disorder. The pandemic also influenced the type of content that people discussed on social media, with users shifting away from “self-focused” perspectives and toward more “other-focused” topics that used to be taboo to discuss [9].

Historically, the availability of mental health treatment services has been inadequate compared with the demand [10]. Mental health care also experiences a paradox of being overdiagnosed yet undersupported, with patients with some symptoms and disorders being readily medicated despite the symptoms and disorders not being understood and accepted socially [11]. Furthermore, many who would benefit from mental health services do not seek or participate in care because they are either unaware of such services, are unable to afford them, or the stigma associated with seeking treatment proves too great a barrier [12]. In fact, two-thirds of people with a known mental disorder do not seek help from a health professional [13].

### Related Work

Many researchers have used social media platforms to explore and understand the dynamics of health care discussions [14]. Several reviews have been carried out on mental health discussion in particular, finding that social media is a viable platform for users to discuss mental health and feel supported, although privacy risks and ethical concerns of the research applications exist as well [15,16]. Previous studies have analyzed the social media content of consenting individuals who have a diagnosed disorder, identifying early markers of depression in Twitter feeds [17,18] and Instagram photographs [19], predicting postpartum depression in Facebook activity [20], and classifying messages from Twitter users self-disclosing various mental illnesses [21,22].

Other studies have analyzed social media feeds of users struggling with mental health more generally, finding that depressed individuals post with higher levels of distorted thinking [23] and identifying markers of suicidal ideation in support threads on Reddit [24] and in messages on Twitter [25]. Several other studies have more directly examined existing social attitudes toward those with mental illnesses, investigating the stigma toward, and treatment of, students with mental illnesses [26,27] and analyzing social media posts that mention various mental illnesses [28-32]. Analysis of text-based crisis-counseling conversations found actionable strategies associated with more effective counseling [33].

Although developments in predicting mental health states provide an opportunity for early detection and treatment, they come with several ethical concerns, such as incorrect predictions, involvement of bad actors, and potential biases [34]. Social media users also hold negative attitudes toward the concept of automated well-being interventions prompted by emotion recognition [35], and they view emotion recognition in general as invasive, scary, and a loss of their control and autonomy [36].

When it comes to using social media as a real-time source of information and opinion, it should be noted that Twitter’s user base is limited, skewing younger and more politically left leaning than the US population overall. Mental health discourse is also a sensitive, often personal topic that many individuals will avoid discussing publicly. Although tweets will fail to capture many aspects of human behavior, estimates of public opinion based on the tweets can complement survey-based measures. Twitter is a valuable social ecosystem from which we can sketch a rough portrait of the existing conversation around mental health, and given that social media lowers the barrier for individuals to join difficult conversations, especially with Twitter allowing users to sign up anonymously, it is a promising source of unstructured language data describing the changing experience of a stigmatized group.

### Objectives

Although stigma has proven to be a significant barrier to receiving treatment from formal (eg, psychiatrists and counselors) and informal sources (eg, family and friends), the COVID-19 pandemic and the associated isolation, grief, and hardships have spurred awareness of mental illness and discussion on this topic in public forums such as social media. By measuring changes in this conversation, we aim to quantify the hypothesized increase in discussions and awareness and the corresponding reduction in stigma around mental health. Using messages from Twitter, we examine the growth of public attention on mental health, the divergence of language from general messages and their associated happiness shifts, and finally the rise of ambient words or phrases. With these measurements, we can piece together how this topic and its social attention has shifted in the past decade.

## Methods

### Data

The source of data for this study is the Decahose application programming interface by Twitter, filtered for English messages, from which we collected a 10% random sample of all public tweets between January 2010 and January 2021. This collection was separated into three corpora consisting of (1) all tweets, (2) tweets containing the phrase *mental health*, and (3) tweets containing a small set of phrases related to mental health. Statistics and time series comparisons among the corpora were carried out as described in the following sections.

### N-Grams

#### General Twitter

To explore trends in the appearance of words, we processed messages from January 2010 through January 2021 into 1-, 2-, and 3-grams, where a 1-gram is a 1-word phrase, a 2-gram is a 2-word phrase, and so on, using the n-gram popularity data set StoryWrangler [37].

For each day, we counted the number of times each unique n-gram appeared in tweets and determined use frequencies compared with the appearance of other phrases on Twitter. We ranked n-grams by descending order of count; n-grams with a low rank value assigned to phrases appear on Twitter very often, whereas those with a high rank value appear rarely. For example, the 1-gram *a* has a median rank of 1 because it is typically the most commonly used word in the English language, whereas the 1-gram *America*, which is less common, has a median rank of 990 [38]. To better visualize this concept of descending count in the figures presented in this paper, we plotted rank on an inverted axis.

#### Mental Health Collection

To explore the specific language used when discussing mental health on Twitter, we compiled a separate collection of n-grams from tweets related to this topic from the same time frame. Restricting the list to messages from 2010 through 2021 that contained the 2-gram *mental health*, we created n-grams in the same fashion as previously described, determining their use frequency within this anchor set and ranking phrases by descending order of counts. We also computed the aggregated frequency and rank of n-grams over each year, using the existing count values for each day, summing them over each year, and ranking them by these counts. Summary statistics for several of the key events in this new data set compared with the general 1-gram data set are shown in Table 1, which highlights the size of the mental health collection over the years. In 2012, roughly 1 in 10,000 messages referenced mental health, whereas in 2018, the rate was roughly 1 in 100 messages. Even so, the mental health collection remains a small subset of messages compared with Twitter as a whole.

**Table 1:** Summary statistics of the mental health n-gram data set compared with the general Twitter n-gram data set on 3 individual days. The dates shown correspond to several Bell Let’s Talk Day events occurring between 2010 and 2021. Bell Let’s Talk Day is an annual fundraising and awareness campaign in Canada that coincides with the annual peak in conversation regarding mental health. Unique 1-grams enumerate the set of distinct words found in tweets on these dates, reflecting roughly 10% of all tweets. The Total 1-grams column shows the sum of the counts of each unique 1-gram, and the Total 1-grams (no retweets) column shows the sum of the counts of 1-grams in tweets, not including any messages that were retweeted.

			Unique 1-grams		Total 1-grams		Total 1-grams (no retweets)
**February 8, 2012**							
	Mental health	3.0×10^3^		3.0×10^4^		9.3×10^3^	
	General	1.7×10^7^		3.1×10^8^		2.2×10^8^	
**January 21, 2014**							
	Mental health	1.6×10^3^		2.3×10^4^		1.5×10^5^	
	General	2.4×10^7^		4.9×10^8^		2.9×10^8^	
**January 31, 2018**							
	Mental health	4.9×10^4^		4.4×10^6^		2.6×10^5^	
	General	2.1×10^7^		5.4×10^8^		1.6×10^8^	

Using these data sets, namely counts of phrases in all tweets (general) versus counts of phrases in tweets containing *mental health*, we analyzed changes in the conversation surrounding mental health over time. The dynamics of several other phrases related to mental health were analyzed as well, but we focused primarily on *mental health* as a representative example of such phrases rather than attempting to exhaustively gather all related content.

## Results

### Growth of Collective Attention

#### Mental Health Discourse

**Figure 1:**
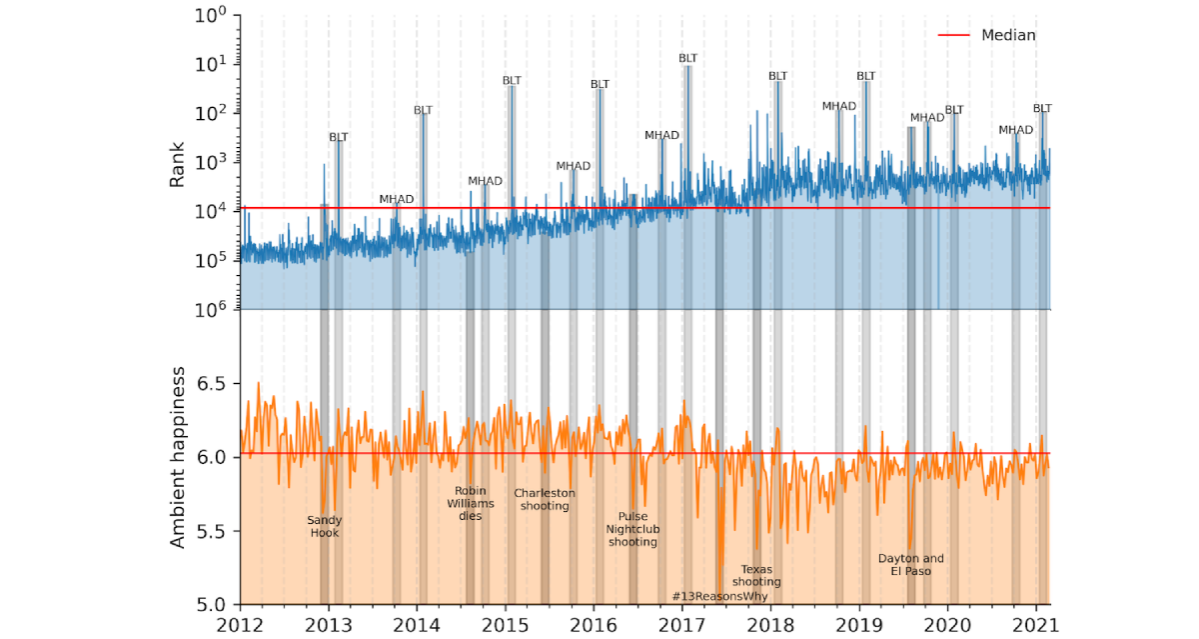
Timeline of mental health discourse on Twitter. The top panel shows the rank time series of the 2-gram mental health over the past decade on a logarithmic axis. Rank is determined by ordering 2-grams in descending order of counts for each day and is plotted on an inverted axis. The median rank value of the time series is highlighted by a horizontal red line. The bottom panel shows the ambient happiness of all messages containing the 2-gram mental health for each day over the same time period. For clarity, these data are shown as a weekly rolling average, and again the median is highlighted by a red horizontal line. Across both panels, key dates are highlighted in gray and annotated with the associated event. These are dates that led to large spikes or drops in either time series. Annually occurring events such as Bell Let’s Talk (BLT) Day or Mental Health Awareness Day (MHAD), are shown in light gray, and unexpected events are highlighted in a darker gray.

Public awareness and education regarding an issue is an important step in reducing negative attitudes because a major component of stigma is lack of knowledge [12]. To understand the general public’s level of awareness of mental health issues, we quantified the frequency at which people on Twitter have discussions about the topic of mental health. Using Twitter n-gram data, we constructed a rank time series of the 2-gram *mental health* on a logarithmic axis, which we have presented in Figure 1.

We find that this 2-gram increased in rank by nearly two orders of magnitude between 2012 and 2018, reflecting a dramatic increase in the discussion of mental health on Twitter. For the first 4 years, only a handful of dates resulted in ranks for *mental health* that were more popular than the overall median, whereas for the final 4 years, only a few dates resulted in ranks indicating less attention than the median.

We also examined the positivity of this conversation, calculating the *ambient happiness* score of messages mentioning the phrase *mental health* for each day, which is also shown in Figure 1. Ambient happiness scores for each day were computed by averaging the scores of each word that appeared in a message with *mental health* for a given day, using the Language Assessment by Mechanical Turk dictionary [39]. Although the rank of this 2-gram has increased over the past decade, the ambient happiness of these messages has decreased since 2017.

Examining the daily behavior of these time series, several dates emerged where either the rank or ambient happiness deviated largely from its baseline behavior. In Figure 1, key events associated with large spikes or drops in the time series are highlighted across both panels. Awareness events such as Bell Let’s Talk Day and Mental Health Awareness Day contribute to the large, annual spikes in rank beginning in 2013. The 2-gram *mental health* reached its highest rank ever on Bell Let’s Talk Day in 2017, peaking at the 18th most popular phrase compared with all other 2-grams on Twitter that day.

Other spikes in rank, and concurrent drops in ambient happiness, occurred on dates with national tragedies such as mass shooting events or celebrity deaths. The largest drop in ambient happiness occurred in 2017 after the deaths of multiple teenagers that were connected to the Netflix series *13 Reasons Why* [40,41].

Looking further into the language used on these specific dates, we show the top 2-grams found in messages containing *mental health* in Multimedia Appendix 1. These co-occurring n-grams are shown with their use rate, rather than rank, so that we can visually see how phrases are being used compared with others in the same list. For example, a popular article shared on December 14, 2012, contained the phrase “It’s currently easier for a poor person to get a gun than it is for them to get treatment for mental health issues.” This phrase was subsequently quoted by thousands of accounts on Twitter [42]. The resulting phrases (Multimedia Appendix 1) provide more insight into what the broader conversation around mental health looked like after these events.

#### Happiness Word Shifts

**Figure 2:**
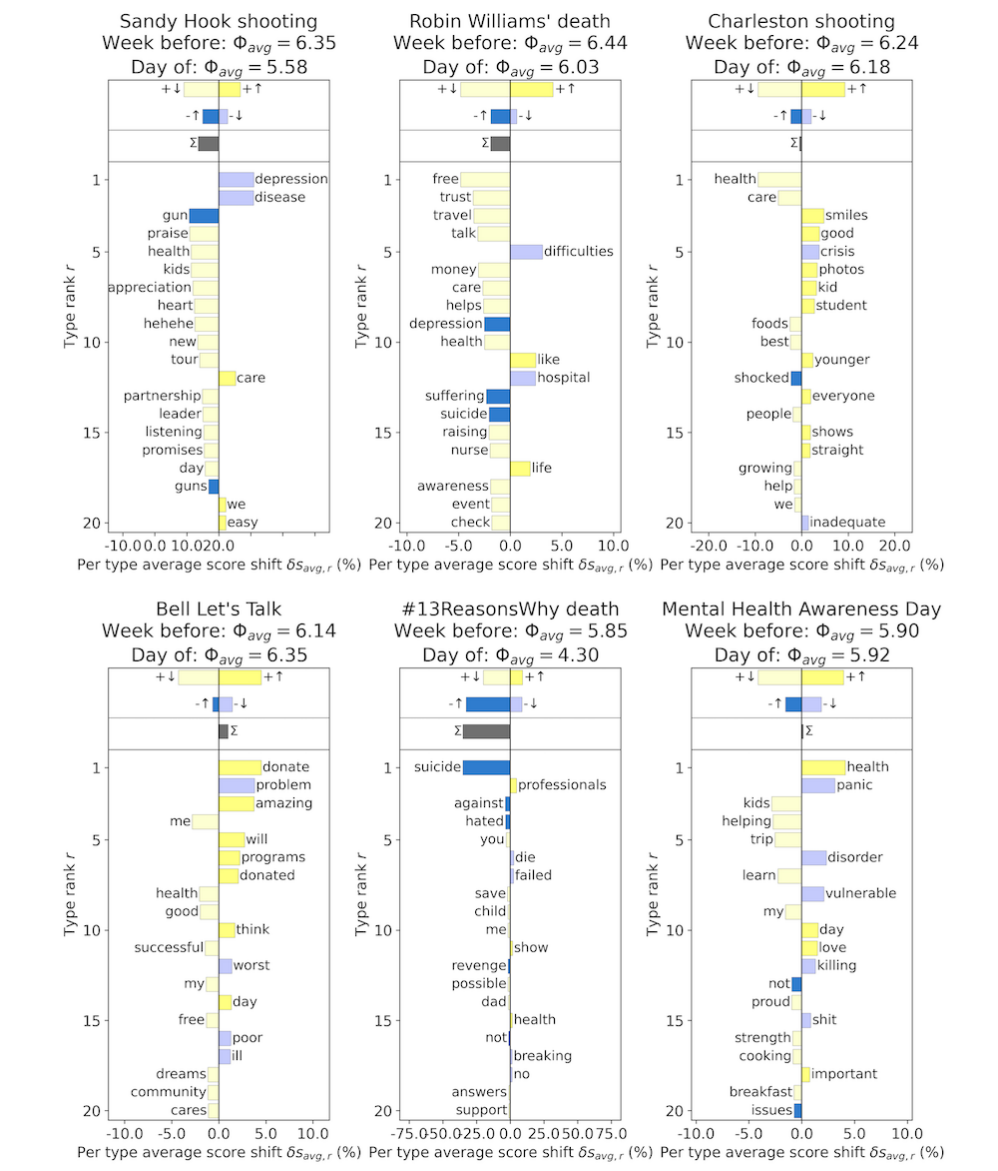
Happiness word shift graphs. In each of the 6 panels, of the 1-grams, we show the 20 that contribute most to the shift in ambient happiness on key dates, compared with the prior week. The words shown in blue are the ones that have been labeled as relatively negative, whereas the words shown in yellow are the ones that have been labeled as relatively positive [43]. The darker shade of these colors tells us where there is an increase in these words, whereas the lighter shade represents a decrease in use. The happiness score shift is shown on the horizontal axis, representing how positive or negative the language on these days becomes, and the happiness rank of the 1-gram in this subset is shown on the vertical axis. Average ambient happiness scores for the day of the event, as well as a week before the event, are also noted at the top of each subplot.

To understand the rise and fall of the ambient happiness scores over the time series in Figure 1, we can look at the words that most heavily contributed to these shifts [43]. Figure 2 highlights words associated with the same key events shown in Multimedia Appendix 1, using messages from a week before the event as a reference. Words highlighted with a blue bar are ones that have been coded as negative, and words highlighted with a yellow bar are ones that have been coded as positive. The darker shades of these 2 colors represent words that have increased in use compared with the reference, whereas lighter shades represent words that have decreased in use. The left side of these panels shows words that are lowering the average score, either through an increase in negative words or a decrease in positive words, and the right side shows words that are raising the score. The average ambient happiness scores for the day of the event and a week before the event are also highlighted at the top of each panel. The 1-grams are also ordered by rank from top to bottom, as shown by the vertical axis.

Looking at Figure 2, we see that mass shooting events have an increase in negative words such as *gun*, *guns*, and *shocked* and a diminishing use of negative words such as *depression*, *disease*, and *crisis*. The day of the Sandy Hook shooting saw fewer positive words such as *praise*, *appreciation*, and *listening*, which would usually be seen in the daily mental health content on Twitter.

Although the Charleston shooting saw a decrease in words such as *health* and *care*, it also saw an increase in positively coded words such as *smiles*, *kid*, and *student*, which likely refer to the shooter in this event. The middle panels in both rows highlight word shifts after death-by-suicide tragedies, and these include an increase in the words *depression*, *suffering*, and *suicide*, which explain the drops in ambient happiness seen on these days.

The awareness events Bell Let’s Talk Day and Mental Health Awareness Day, which represent the only increases in ambient happiness on the dates shown in Figure 2, both show an increase in quite a few positive words: *donate*, *amazing*, *programs*, *health*, *love*, and *important*. These days also notably see a decrease in strongly negative words such as *problem*, *disorder*, *vulnerable*, and *killing*.

### Narrative and Social Amplifications

#### Rank-Turbulence Divergence

The increasing appearance of the phrase *mental health* could be due to several factors. We analyzed the corpus associated with the topic of *mental health* using the n-grams and their relative frequency and rank values for each day and compared the word use in this subset with a random sample of messages on Twitter.

To compare differences in language use, we used rank-turbulence divergence [44]. With this method, we could examine the shift in language between the 2 samples of tweets. We aggregated n-gram counts for phrases found in tweets containing *mental health* over the span of each year, getting annual counts for each of these phrases.

**Figure 3:**
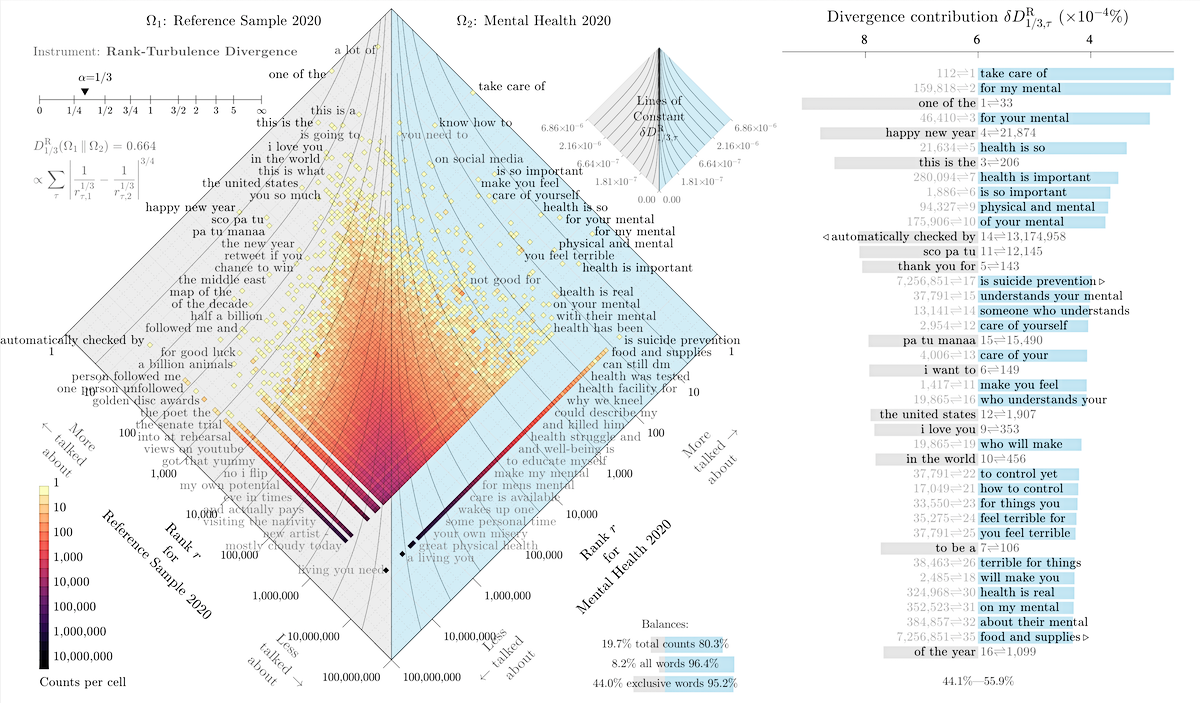
Allotaxonograph using rank-turbulence divergence of 1-grams from tweets in 2020 containing the anchor phrase mental health compared with a random sample of tweets in 2020. In the central 2D rank-rank histogram panel, phrases appearing on the right have higher rank in the mental health subset than in random tweets, whereas phrases on the left appeared more frequently in the random sample. The table to the right shows the words that contribute most to the divergence. Note that when ranking 3-grams from mental health tweets, * mental health and mental health * phrases were removed for clarity. The balance of the words in these 2 subsets is also noted in the bottom right corner of the histogram, showing the percentage of total counts, all words, and exclusive words in each set. See Dodds et al [44] for a detailed description of our allotaxonometric instrument.

We performed the same aggregation for a smaller random subset of Twitter data, aggregating yearly data for a 1% sample of the Decahose application programming interface. Figure 3 highlights the results of rank divergence comparing the 2 subsets of messages across 2020. When ranking 3-grams from mental health tweets, * mental health and mental health * phrases were removed for clarity.

Each square histogram bin reflects the relative ranks for 3-word phrases in each respective subset. Bins to the right side contain 3-grams with relatively higher rank in the right subset than in the left. The bins down the middle of the plot contain words with a similar rank in both subsets. The bands of bins on the bottom edges of these plots represent words that are exclusive to their respective side’s data set.

The color of each bin correlates with the density of words contained in it, and the words appearing on the plot are randomly selected representatives from the bins on the outer edges. The table on the right shows the words that contribute most to the divergence of the 2 data sets, with small triangles indicating when a word is exclusive to a system. For example, the phrase *take care of* was the 112th most common 3-gram in random tweets posted during 2020, but it was the most common 3-gram in tweets containing *mental health*.

When comparing n-grams from these subsets in Figure 3, we see that the mental health data set, shown on the right side of the figure, includes language related to taking care of one’s physical and mental health, suicide prevention, men’s mental health, social media, and personal time. Although we would expect to see pandemic-related phrases show up in 2020, these topics were equally mentioned across both samples; therefore, they do not appear on either side of this histogram.

#### Contagiograms

To better understand the dynamics of phrases related to mental health, we explored ways in which these messages were spreading across Twitter. Tweets can be posted as original content in a new message or a user can retweet a message that another user has posted.

Organic messages show that users are writing their own content related to a topic, whereas retweeted messages show that this topic is being shared and spread to other groups of users; both are important means of contributing to the conversation. Both organic messages and retweeted messages appear in our data set and are included in the previous analyses; therefore, it is important to also examine the proportion of messages that fall into these 2 categories.

**Figure 4:**
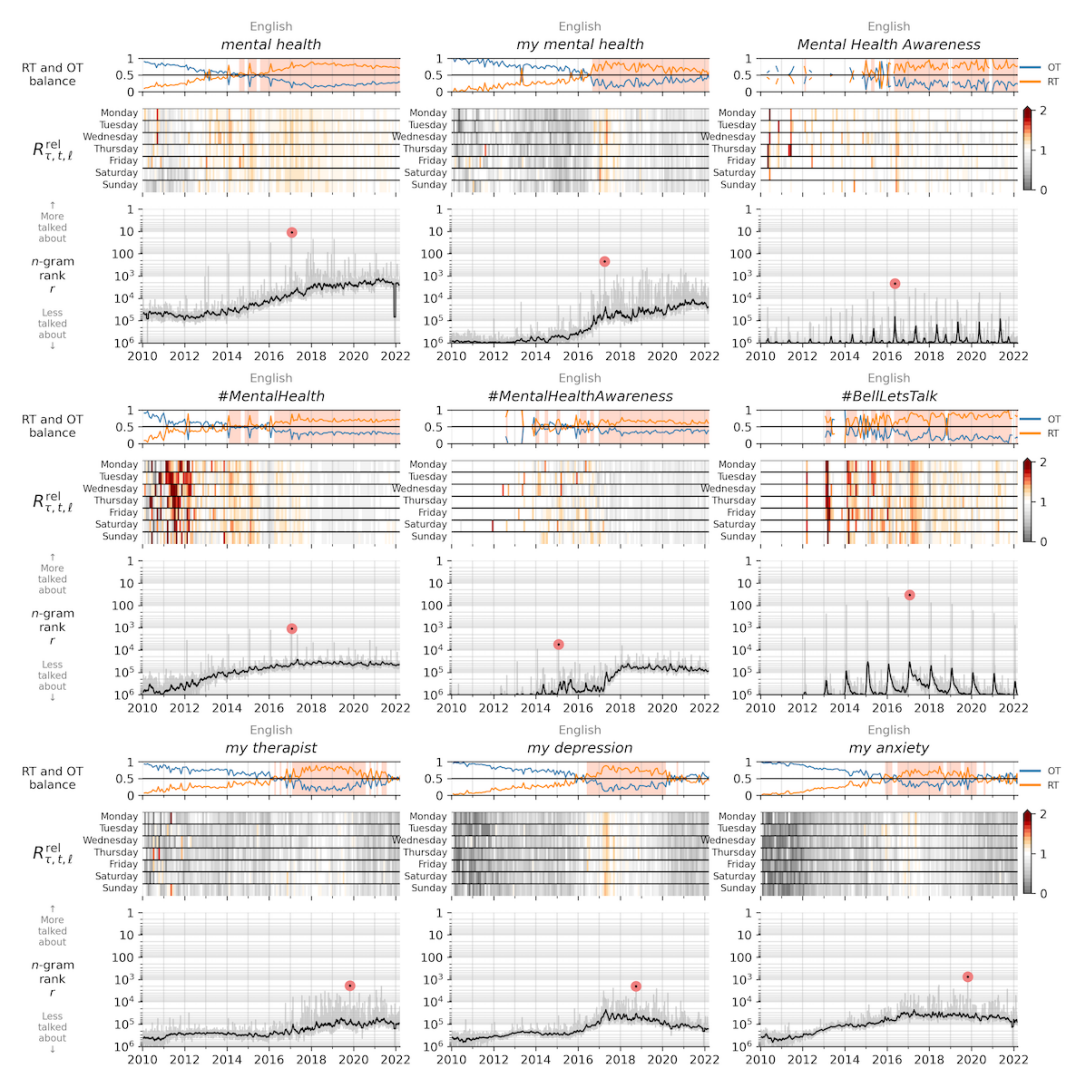
Contagiograms for mental health–related n-grams. In each subplot, the top panel displays the monthly relative use of each n-gram, indicating whether they appear organically in new tweets (organic messages [OTs], blue) or in shared retweets (retweeted messages [RTs], orange). The shaded area highlights time frames when the number of RTs is higher than that of OTs, suggesting social amplification [37]. The middle panel of each subplot shows the retweet use of each n-gram compared with the background rate of retweets among all English tweets, with a heatmap for each day of the week. For these heatmaps, the color map is shown on the right, with darker red representing a higher relative rate of RTs among these messages compared with general messages and gray representing a higher rate of OTs. The bottom panel shows the basic n-gram rank time series, with a month-scale smoothing of the daily values shown in black and background shading in gray between the minimum and maximum ranks of each week. Note that phrase counts only reflect tweets that have been identified as messages written in English as discussed by Alshaabi et al [45].

Figure 4 shows *contagiogram* plots, as implemented by Alshaabi et al [45], that highlight the relationship between retweeted and organic content for a given n-gram on Twitter. The top panel of these plots shows the monthly relative use of the specified n-gram, highlighting the use of organic messages in blue and shared retweets in orange. A shaded area in this top panel represents time periods when the number of retweeted messages surpasses that of organic messages, highlighting social amplification.

The middle panel shows retweet use of an n-gram compared with the rate of all retweeting behavior across English Twitter, using a heatmap for each day of the week across the time series. In this heatmap, darker red shades represent a higher relative rate of retweets for the given n-gram compared with a random English n-gram on Twitter and gray shades represent a higher rate of original messages. The bottom panel provides the rank time series of the n-gram, with a month-scale smoothing of the daily values shown in black. In Figure 4, we look at these contagiogram plots for a collection of key n-grams related to the discussion of mental health on Twitter.

Across each of the subplots in Figure 4, we see that phrases and hashtags related to the topic of mental health have grown in volume throughout the time period studied, as reflected by their popularity compared with all tweets. Looking at English Twitter overall, the balance of messages tilted toward primarily organic until mid-2017, when the practice of retweeting messages tipped the balance [45]. Around this same time, retweeted messages reached higher numbers than organic messages for most mental health–related n-grams, as seen in the top panels of these subplots.

Examining the heatmap panels of these subplots, we observe a larger social amplification effect in hashtags related to mental health, highlighted by the darker red shades across the heatmaps. However, in recent years, these hashtags shifted to more organic messages, with the heatmaps becoming more gray after 2018. The hashtag *#BellLetsTalk* sees the most retweeted behavior of these hashtags, as well as an annual spike on the day of the event, followed by a substantial tail of conversation after this date. On Mental Health Awareness Day (October 10) in 2018, organic tweets referencing #BellLetsTalk spiked, leading to the inversion of retweeted messages and organic messages in late 2018 that we see in Figure 4. We also see more original content containing self-disclosure phrases such as *my therapist* or *my depression*, as seen in the third row of n-grams that have largely gray shades across the heatmaps.

## Discussion

### Principal Findings

In this project, we explored the conversation around mental health and its appearance on the social media platform Twitter. Using a collection of phrases, we examined how often the topic of mental health was discussed in tweets, finding that the 2-gram *mental health* has increased in rank by nearly two orders of magnitude since 2012. We calculated the associated ambient happiness for the same time series, finding that happiness is largely affected by key dates and has generally decreased over the past decade. After compiling a new data set of n-grams found in the subset of tweets mentioning *mental health*, we analyzed text associated with this specific term, finding the top n-grams related to the topic and their use rates. We examined the language in this conversation across years, finding topics that emerged over the past year since the pandemic began. Comparing the use rates of retweeted content and original content, we found that common *awareness* messages were being amplified on the social media platform, whereas personal self-disclosing statements were being seen more in organic, originally authored content. These results provide valuable insight into how the discussion of mental health has changed over time and suggest that more awareness and acceptance has been brought to the topic compared with past years.

### Growth of Collective Attention

#### Mental Health Discourse

Our findings suggest that the number of mental health conversations on Twitter has substantially increased in recent years, particularly on dates associated with either awareness campaigns or tragedies. Several dates across the time series emerge where either the rank or ambient happiness deviates largely from its baseline. Awareness events such as Bell Let’s Talk Day and Mental Health Awareness Day contribute to the large, annual spikes in rank beginning in 2013. Bell Let’s Talk Day, falling on the last Wednesday of January each year, was started by the Canadian company Bell Telephones and aims to bring awareness to the general public about mental health issues by donating CAD $0.05 (US $0.04) for each tweet using its hashtag *#BellLetsTalk*. Other spikes in rank, and concurrent drops in ambient happiness, occurred on dates with national tragedies such as mass shooting events or celebrity deaths. The largest drop in ambient happiness occurred in 2017, immediately after the death of a teenager that was connected to the Netflix series *13 Reasons Why* [39]. Looking at the events that sparked more conversations around the topic of mental health, and their associated levels of ambient happiness, awareness campaigns tended to lead to a rise in ambient happiness, whereas unexpected events, of which all would be considered tragedies, led to a drop in ambient happiness.

#### Happiness Word Shifts

Looking at the words most heavily contributing to the shifts in sentiment on these dates, we found that although mass shooting events see an increase in negative-coded words related to gun violence, this can sometimes coincide with positive-coded words related to students and children. This example highlights the drawbacks of dictionary-based ambient happiness analysis without context of the words being used because independently positive words can be used to describe a tragic event.

The word shift graphs in Figure 2 also highlight the drop in ambient happiness after death-by-suicide tragedies, which see an increase in words related to depression and suffering. We found that awareness days represent the only increase in ambient happiness on these dates, with an increase in words related to donating, health programs, and love. These awareness days also see a notable decrease in many strongly negative–coded words. These results highlight the shift in language on awareness days, away from phrases with negative connotations and focusing on language relating to community support and aid.

### Narrative and Social Amplifications

#### Rank Divergence

When comparing n-grams from the mental health subset and random Twitter subset, we see that the mental health data set includes language related to taking care of one’s physical and mental health, suicide prevention, men’s mental health, social media, and personal time. These topics seem to have become more prominent in 2020, with people being at home and isolated during the COVID-19 pandemic and with more awareness being brought to the relationship between social media and mental health.

Studies in 2020 have shown that at the onset of the pandemic, Google searches for terms related to mental health increased initially, followed by a *flattening out* after stay-at-home orders were announced [5]. It has also been recorded that between March 2020 and July 2020, average phone screen time doubled to 5 hours per day and rates of depression increased by 90% [4]. Although these figures cannot tell us everything about how language differs among subsets of conversation, they do provide a sense of the mental health topics individuals discussed in 2020.

#### Contagiograms

Comparing the use rates of retweeted content and original content, we found that common *awareness* messages are being amplified on the social media platform, whereas personal self-disclosing statements are being seen more in organic, originally authored content. These relationships suggest that users are sharing hashtags to spread awareness and they feel comfortable retweeting hashtags posted by others. The public disclosure of private personal anecdotes, which helps to normalize conversation about personal struggles with mental health, is treated differently. Overall, our results suggest that a larger number of individuals feel comfortable making mental health disclosures publicly, but they are amplified relatively less often than other types of mental health messages.

We also see a substantial increase in the ranks of all phrases and hashtags related to mental health over time, with annual awareness days resulting in spikes corresponding to their given date each year. These findings offer evidence that an understanding of mental health conversations has increased substantially over time, reducing the stigma surrounding mental illness.

### Limitations

We acknowledge that using Twitter as a data source for this research has many limitations because its user base is not a broadly representative sample of the human population, and thus these messages will fail to capture many aspects of human behavior. A study by the Pew Research Center [46] shows that as of June 2019, only 22% of all US adults reported using Twitter, smaller than, for example, the 69% who use Facebook. The age breakdown of users is also skewed, with 38% of individuals aged 18-29 years using Twitter, whereas only 17% of those aged 50-64 years use the site. Although demographics of race are fairly uniform (21% of White adults, 24% of Black adults, and 25% of Hispanic adults), the platform is more often used by individuals with a college degree (32%) and living in an urban area (26%) [46].

Mental health discourse is a sensitive and personal topic that many individuals avoid discussing publicly. However, social media has the ability to lower the barrier for individuals to engage in difficult conversations because Twitter allows users to both sign up anonymously and retweet messages in addition to writing their own messages. This being said, we recognize that a portion of Twitter accounts are run by businesses, institutions, and other organized groups, rather than simply individuals. These corporate accounts, such as *@Bell_LetsTalk*, would have more of a pattern and agenda to their posted tweets, and there is not currently a way to filter out these messages. Because of these complexities of the Twitter user base, care must be taken when interpreting findings based on tweets.

These limitations could be addressed in future studies by expanding the data sources; for example, by looking to other available websites such as Reddit, Instagram, or Facebook, whose user bases differ in some aspects. Turning away from social media, one could examine clinical records for cases of diagnosed mental illness, analyzing the language and positivity of physician notes. Rather than looking at simply the messages of this social media platform, this work could be expanded to address the conversation on a network scale, determining how interactions among users affect the discourse.

This study is also limited to the anchor phrase *mental health* and thus could be leaving out conversations related to the topic. To further enrich these findings, future work could expand the existing mental health data set to include tweets with additional anchor n-grams, although a method for determining these anchors would be necessary.

Our results are also limited to the English language and thus also to events occurring in English-speaking regions. The mass shooting events noted in this study are specific to the United States, and the television show *13 Reasons Why*, although available internationally, led to reports in the United States of an increase in deaths by suicide among teenagers. Although several of the events noted may be specific to the United States, these were events that were discussed heavily across all of English-speaking Twitter and the trends we found relating to awareness campaigns, celebrity deaths, and the pandemic’s effect on mental health can be generalized to other regions experiencing these or similar events.

### Conclusions

We believe that the results presented here provide useful texture regarding the growing conversation around mental health on Twitter as well as evidence that more people are contributing to this conversation on the social media platform than ever before. Our findings suggest that the number of conversations around this topic have substantially increased on Twitter in recent years and spike especially high on dates coinciding with events such as awareness campaigns, television series releases, mass shootings, and celebrity deaths. These events also drastically shift the ambient happiness associated with the topic of mental health during these time periods. Awareness campaigns positively drive the ambient happiness, as well as shift the focus away from negative connotations and toward the importance of community care, support, and aid, whereas the tragedy events lead to a drop in ambient happiness because they see a focus on suffering, gun violence, and death by suicide. When comparing the mental health data set to a control sample of Twitter users, topics emerge around suicide prevention, taking care of one’s mental health, social media, and personal time, all of which became more prominently discussed in 2020. Awareness messages are heavily amplified on the platform through retweets, and personal self-disclosure statements are being posted in more originally authored content.

As mental health becomes talked about more, and awareness campaign efforts seem to be driving a large portion of this increase, public health campaigns aiming to reduce stigma surrounding mental health can leverage this information to improve their messaging. The knowledge that young people on Twitter are participating in these conversations, whether through retweets or personal statements, shows the role social media could have in spreading this conversation to other users in an effort to normalize mental health and reduce the stigma surrounding it.

We also learn from these results that some tragic events, such as mass shootings, bring up interesting conversations related to the link between gun violence and mental health and how much of these horrific events is attributed to the mental illness of the offender. These conversations are complicated and have the potential to not only bring light to the need for better mental health care but also further the stigma around mental illness while avoiding the debate around gun violence as an issue on its own. Knowing that there is a documented link among these conversations after their associated events, perhaps we can inform further debates on, and policy decisions for, these issues.

Finally, we find that television shows can have devastating impacts if their content, portrayal, and significance are not well thought out before creation. Studios, directors, and streaming companies all have a responsibility, especially with projects aimed toward younger audiences, to properly screen their content and think deeply about the impact of each choice that they make. Policies around these safety concerns, if they do not exist already, should be put into place to avoid future tragedies linked to this effect.

As this conversation on the topic of mental health continues to grow, and perhaps becomes more normalized, it will be useful to examine the language associated with future events and how it shifts over time.

## Appendix

Multimedia Appendix 1 Top n-grams used in discussions on mental health during spike dates.
