# Supplementary material for: Quantifying Changes in the Language Used Around Mental Health on Twitter Over 10 Years: Observational Study
Source: JMIR Ment Health. 2022 Mar 30;9(3):e33685. doi: 10.2196/33685 (PMC9008521; doi:10.2196/33685)
Supplement: Multimedia Appendix 1 [file mental_v9i3e33685_app1.pdf]

Multimedia Appendix

Top n-grams used in discussions on mental health. Here, of the 2-grams that appear in the mental health tweet collection for a few outlier dates noted in Figure 1, we show the top 15. Each subplot lists the date and its associated event, along with a bar graph of the use rate. It is worth noting that the bars in each subplots cannot be compared with those of the other subplots because the ranges of the x-axes are varied for clarity.

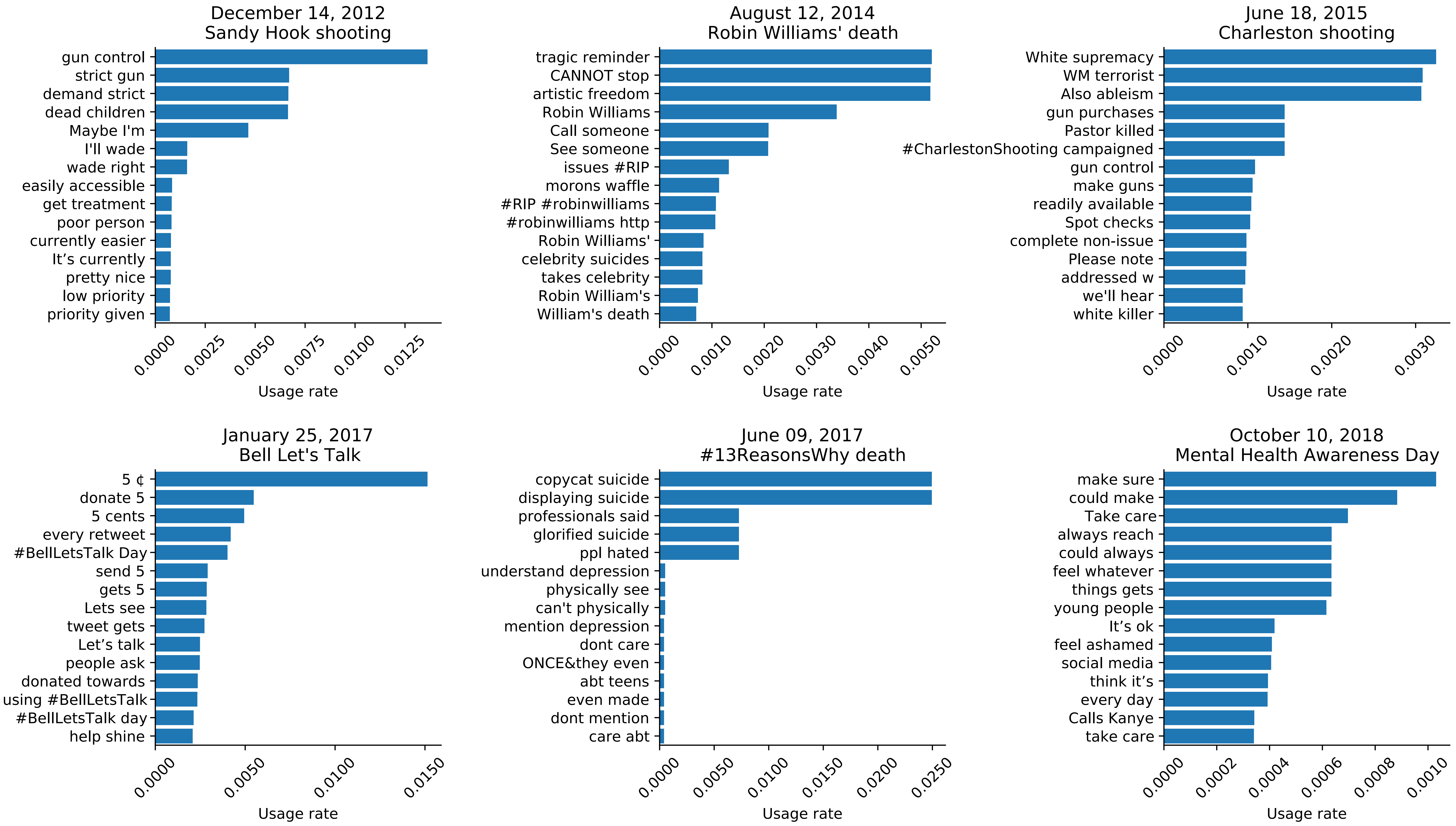

This is a Multimedia Appendix to a full manuscript published in JMIR Mental Health. For full copyright and citation information see https://dx.doi.org/10.2196/33685
